# Progressive Multiple Sclerosis Presenting With Obstructive Uropathy and Acute Kidney Injury: A Case Report

**DOI:** 10.7759/cureus.89643

**Published:** 2025-08-08

**Authors:** Ahmed Jamil, Anup Banerjee, Suham Amin, Nosheen Habib

**Affiliations:** 1 Acute Medicine, Stepping Hill Hospital, Stockport NHS Foundation Trust, Stockport, GBR

**Keywords:** demyelinating disorder, multiple sclerosis, obstructive uropathy, progressive multiple sclerosis, very late onset multiple sclerosis

## Abstract

Very-late-onset multiple sclerosis (VLOMS), defined as disease onset after the age of 60, is a rare and often diagnostically challenging entity that may present with atypical features. We describe the case of a 67-year-old man who presented with progressive urinary symptoms culminating in obstructive uropathy and acute kidney injury (AKI), ultimately diagnosed as progressive multiple sclerosis (MS). The patient had a three-year history of left upper limb weakness and gait difficulty, which had been previously unexplored. He presented acutely following a fall, with new-onset left-sided facial droop and worsening lower limb weakness. Laboratory investigations revealed severe hyperkalemia (serum potassium: 9.8 mmol/L), uremia, and elevated creatinine (731 µmol/L), indicating significant renal impairment (estimated glomerular filtration rate (eGFR): 7 mL/min/1.73 m²). Urinary tract imaging revealed bilateral hydronephrosis and a trabeculated bladder, suggestive of chronic urinary retention; the prostate was enlarged but without significant prostate-specific antigen (PSA) elevation. Catheterization led to immediate bladder decompression, and the patient received medical treatment for hyperkalemia. Neuroimaging (MRI brain and spine) revealed multifocal demyelinating lesions involving periventricular, temporal, pontine, and cervical cord regions, while a lumbar puncture confirmed the presence of oligoclonal bands in both CSF and serum. Neurological examination demonstrated upper motor neuron signs, including facial asymmetry, limb spasticity, and pyramidal weakness, further supporting a central nervous system etiology. Despite the presence of benign prostatic hyperplasia (BPH) and cervical spondylosis, the degree of neurological impairment, distribution of MRI lesions, and cerebrospinal fluid analysis collectively pointed to a diagnosis of progressive MS with neurogenic bladder dysfunction. This led to urinary retention, obstructive uropathy, and subsequent AKI. While lower urinary tract dysfunction is a common complication of MS, its initial manifestation as acute renal failure is rare, especially in patients without a prior diagnosis. This case highlights the diagnostic complexity in elderly patients where structural (BPH, spinal stenosis) and neurological causes may overlap. It also underscores the importance of a high index of suspicion for demyelinating disease in patients with unexplained bladder dysfunction, progressive motor deficits, and renal impairment. Early multidisciplinary involvement, including neurology, urology, nephrology, and rehabilitation, is essential for prompt diagnosis, bladder decompression, and prevention of irreversible renal damage. Long-term catheterization was instituted, with outpatient follow-up arranged to assess suitability for clean intermittent self-catheterization and continued neurological monitoring. This case illustrates that in older adults, especially men, attributing urinary symptoms solely to common urological conditions may overlook more insidious neurologic diseases such as MS. Timely recognition and appropriate intervention can significantly alter prognosis by preserving renal function and optimizing functional outcomes.

## Introduction

Multiple sclerosis (MS) is a chronic, immune-mediated demyelinating disorder of the central nervous system (CNS), most commonly presenting between the ages of 20 and 40 [1]. Late-onset MS (LOMS), defined as disease onset after the age of 50, is relatively uncommon, and very-late-onset MS (VLOMS), with onset after 60 years of age, is particularly rare. These late presentations are often associated with atypical or progressive symptoms that can complicate or delay diagnosis [2,3].

Lower urinary tract dysfunction is a well-recognized but often underappreciated complication of MS, affecting up to 75% of patients during the course of the disease [4]. Symptoms may include urgency, frequency, incontinence, and urinary retention, most commonly due to neurogenic bladder dysfunction. Urodynamic studies frequently demonstrate detrusor overactivity in over 60% of patients and detrusor underactivity or detrusor sphincter dyssynergia in approximately 20% [4,5]. In older adults, these symptoms are often misattributed to more common age-related conditions such as benign prostatic hyperplasia (BPH), urinary tract infections (UTIs), or detrusor overactivity, which may obscure the underlying neurological pathology and delay appropriate evaluation [6].

We present the case of a 67-year-old man who initially presented with urinary symptoms and was found to have bilateral hydronephrosis and severe acute kidney injury (AKI) secondary to urinary retention. Further investigation revealed progressive MS as the underlying cause. While neurogenic bladder is frequently seen in MS, its presentation with obstructive uropathy and renal failure is rare, particularly in newly diagnosed cases. This case highlights the importance of maintaining a broad differential diagnosis for urinary symptoms in elderly patients, particularly when accompanied by unexplained renal impairment or subtle neurologic signs.

## Case presentation

A 67-year-old man presented following a fall, with a background of progressive left upper limb weakness over three years and increasing walking difficulty. Over the preceding few months, he had also developed difficulty initiating micturition and a sensation of incomplete bladder emptying. On the day of admission, he experienced a fall at home and was noted to have new-onset left-sided facial droop along with worsening left lower limb weakness.

On examination, he had left-sided upper motor neuron facial weakness, mild spasticity in the left upper limb, and bilateral lower limb spasticity, more marked on the left. Power was reduced in the left lower limb, with 4/5 strength distally and pyramidal-pattern weakness proximally of 3/5. Deep tendon reflexes were brisk throughout, and vibration sense was diminished at the halluces bilaterally. Initial blood tests revealed a serum sodium of 137 mmol/L, potassium of 9.8 mmol/L, urea of 57.4 mmol/L, and a creatinine of 721 µmol/L, corresponding to an estimated glomerular filtration rate (eGFR) of 7 mL/min/1.73 m² (Table 1).

**Table 1:** Laboratory results for the patient on admission showing the full blood count and kidney function test. eGFR: estimated glomerular filtration rate.

Parameter	Patient value	Reference value
White cell count	12.7 × 10^9^/L	3.7-11.0 × 10^9^/L
Hemoglobin	87 g/L	115-165 g/L
Platelet count	445 × 10^9^/L	150-450 × 10^9^ /L
Neutrophils	10.3 × 10^9^/L	1.7-7.5 × 10^9^ /L
C-reactive protein	44.2 mg/L	0.0-10.0 mg/L
Serum sodium	137 mmol/L	133-146 mmol/L
Serum potassium	9.8 mmol/L	3.5-5.3 mmol/L
Serum urea	57.4 mmol/L	2.5-7.8 mmol/L
Serum creatinine	731 µmol/L	62-115 µmol/L
eGFR	7 mL/min/1.73 m²	

**Figure 1:**
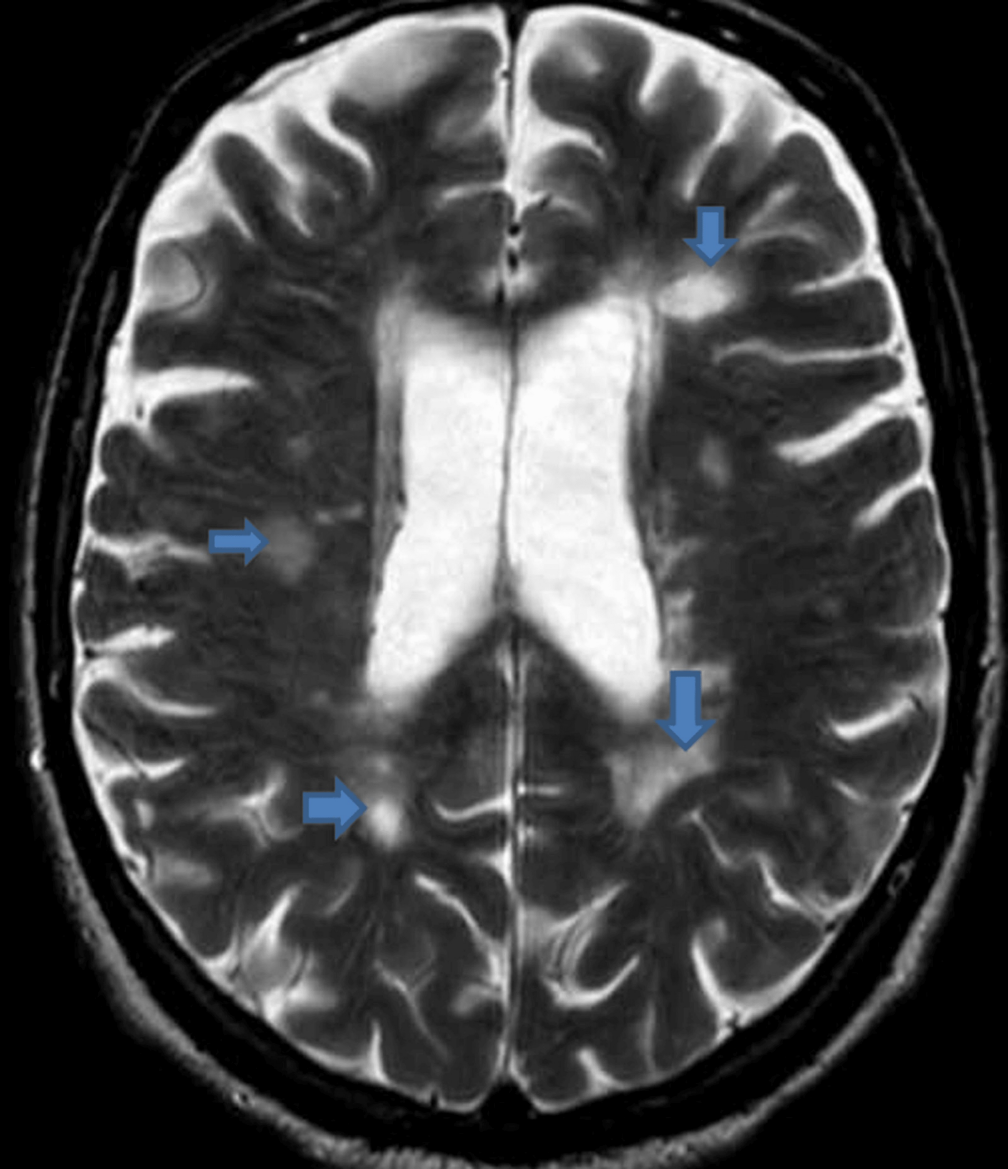
MRI head of the patient, arrows showing multiple demyelinating changes.

MRI head rules out acute stroke but shows confluent periventricular and anterior temporal white matter hyperintensities, as well as involvement of the right cerebral peduncle, raising the suspicion of a demyelinating process (Figure 1).

**Figure 2:**
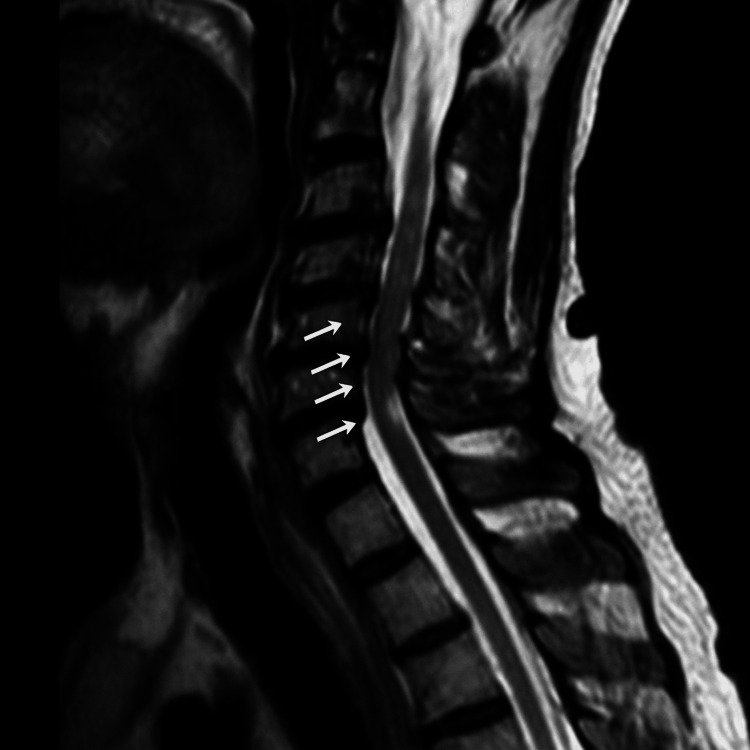
MRI spine showing cervical spondylosis with patchy T2 hyperintensities (arrows).

A CT scan of the urinary tract demonstrated bilateral severe hydronephrosis and hydroureter, along with a thickened, trabeculated urinary bladder with enlarged prostate suggestive of chronic urinary outflow obstruction. The patient was catheterized urgently, managed with hyperkalemia treatment protocols, and referred to the urology team. MRI whole spine was examined that demonstrated multilevel cervical spondylosis with patchy T2 hyperintensities at C3-C6 and associated canal stenosis but no definitive cord compression at any level (Figure 2).

**Figure 3:**
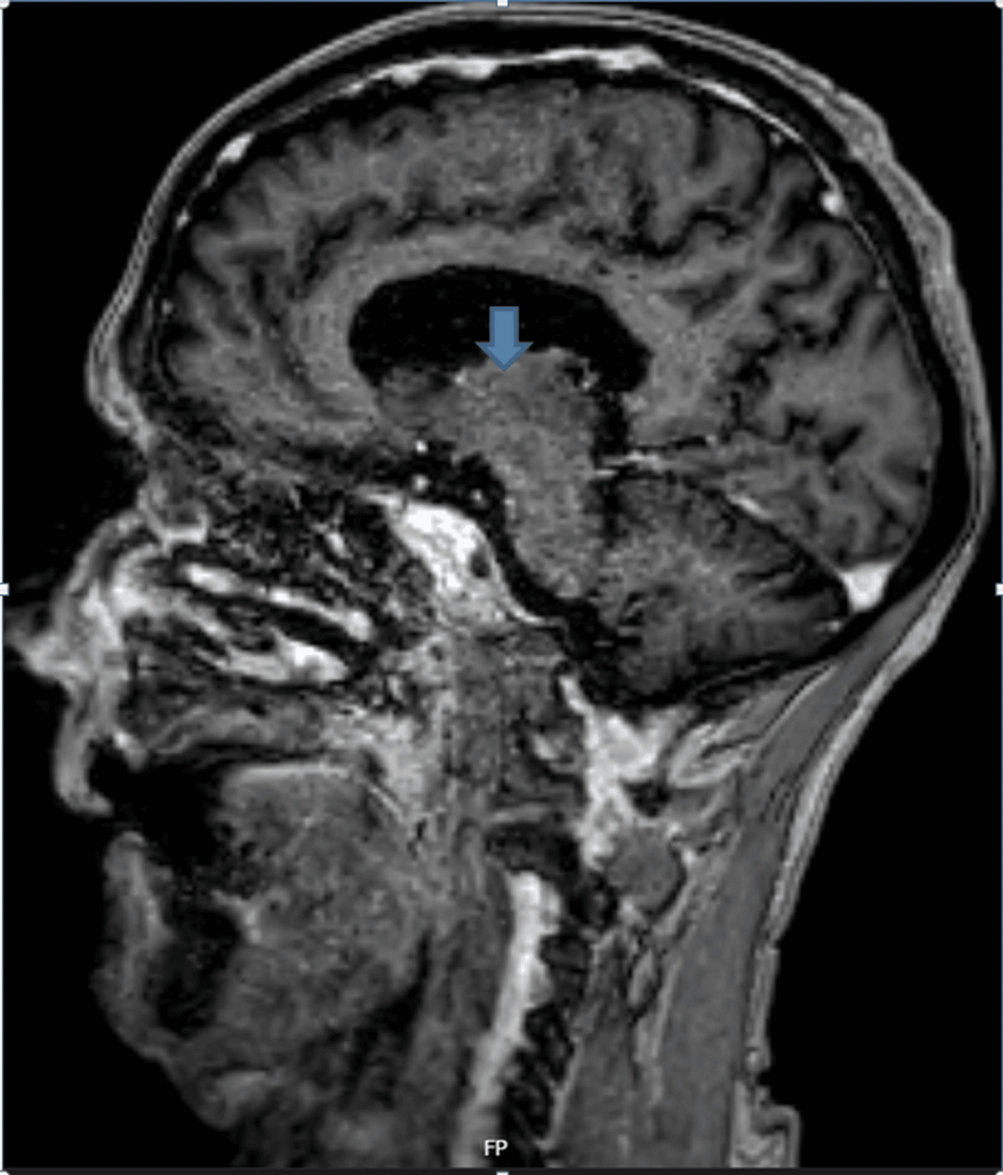
MRI sagittal section showing septocallosal white matter lesion (arrow).

Urology opinion was taken, which advised a prostate-specific antigen test that came back negative, and an outpatient cystoscopy along with outpatient follow-up if symptoms start to improve. Neurology was consulted and advised lumbar puncture and MRI head with contrast and sagittal fluid-attenuated inversion recovery (FLAIR) sequences. Lumber puncture (LP) shows type 3 oligoclonal bands positive (Table 2), and MRI head with contrast and sagittal FLAIR sequences shows multiple septocallosal, deep, and subcortical white matter lesions and one within the right pons. These lesions are highly suspicious for demyelination. There is no abnormal enhancement of any of these lesions (Figure 3).

**Table 2:** Laboratory results for the patient showing lumber puncture result.

Parameter	Patient value	Reference value
Oligoclonal bands	Present in CSF and serum, with additional band present in CSF (type 3)	
CSF total protein	0.39 g/L	0.15-0.60 g/L

Based on the clinical presentation and imaging findings, a diagnosis of progressive multiple sclerosis was made. The patient was discharged with a long-term catheter in place, and outpatient follow-up was arranged with neurology, urology, and spinal services for further management and multidisciplinary input.

## Discussion

Multiple sclerosis (MS) commonly causes neurogenic bladder dysfunction, affecting up to 75% of patients, due to demyelinating lesions that disrupt coordination between the bladder muscle (detrusor) and urethral sphincter [6]. While urgency and incontinence are more frequent, urinary retention arising from detrusor underactivity or detrusor sphincter dyssynergia can occur and may lead to obstructive uropathy and severe renal complications [7].

In the present case, urinary retention occurred because MS-related nerve damage impaired the coordination between bladder contraction and sphincter relaxation [6,7]. Detrusor sphincter dyssynergia (DSD), defined by involuntary sphincter contraction during attempted voiding, leads to functional bladder outlet obstruction [6-8]. This, along with possible detrusor underactivity, resulted in incomplete emptying, bladder overdistension, bilateral hydronephrosis, and ultimately, acute kidney injury [5,7]. Although uncommon, this constellation has been described in MS; neurogenic bladder can mimic mechanical bladder outlet obstruction, causing backpressure on the kidneys [8].

Notably, the CT kidneys, ureters, and bladder (KUB) also revealed indentation of the bladder base by an enlarged prostate, suggestive of benign prostatic hyperplasia (BPH). While this anatomical finding may have contributed to some degree of outflow resistance, the severity of the upper urinary tract compromise, combined with progressive neurological deficits, supports a predominantly neurogenic cause. In elderly men, BPH and neurogenic bladder often coexist, making it essential to consider both structural and neurological contributors. However, in this case, the extensive neuroimaging findings and classic pyramidal signs point toward MS-related detrusor dysfunction as the principal driver of obstruction.

The added presence of cervical spondylotic changes with cord T2 lesions further complicates the picture, as cervical myelopathy, regardless of etiology, can produce similar bladder dysfunction [9]. This overlap underscores the complexity of diagnosing the underlying cause of lower urinary tract symptoms in patients with multifactorial risk factors.

Urinary retention in MS, particularly when due to detrusor underactivity or detrusor sphincter dyssynergia, poses a substantial risk for upper urinary tract deterioration, including hydronephrosis and progressive renal impairment if left unaddressed [6]. Initial management involves prompt bladder decompression, typically via urethral or suprapubic catheterization, along with correction of any associated metabolic disturbances, such as hyperkalemia, as was required in the present case.

Long-term management strategies are guided by urodynamic findings. Patients with detrusor underactivity often benefit from clean intermittent self-catheterization (CISC), which helps prevent chronic retention and reduces the risk of urinary tract infections. In contrast, those with detrusor overactivity may be treated with antimuscarinic agents, beta-3 adrenergic agonists, or intravesical botulinum toxin A injections, depending on the severity and response to initial therapy [10,11].

The prognosis of MS-related bladder dysfunction is closely associated with the disease subtype and extent of neurological involvement. In patients with progressive MS, urinary symptoms tend to worsen over time and are typically less responsive to disease-modifying therapies [7]. Nevertheless, a multidisciplinary approach, including neurology, urology, physiotherapy, and rehabilitation services, can significantly enhance patient outcomes by preserving renal function and improving quality of life. In cases refractory to conservative management, surgical interventions such as bladder augmentation or urinary diversion may be considered [12].

## Conclusions

This case underscores the importance of maintaining a broad differential diagnosis when evaluating urinary dysfunction in older adults. While neurogenic bladder is a common complication of MS, its presentation with obstructive uropathy and acute kidney injury, particularly in the context of very late-onset MS, is rare and easily misattributed to more common urological conditions. Timely recognition of subtle neurological signs and early multidisciplinary involvement are crucial to avoid diagnostic delay, prevent irreversible renal damage, and initiate appropriate long-term management.
